# Neuromodulação Vagal Auricular e sua Aplicabilidade em Pacientes com Insuficiência Cardíaca e Fração de Ejeção Reduzida

**DOI:** 10.36660/abc.20220581

**Published:** 2023-05-02

**Authors:** Sergio Menezes Couceiro, Lucas Bonacossa Sant’Anna, Mariana Bonacossa Sant’Anna, Renata S. Matos Menezes, Evandro Tinoco Mesquita, Fernando Mendes Sant’Anna

**Affiliations:** 1 Universidade Federal Fluminense Cabo Frio RJ Brasil Universidade Federal Fluminense, Cabo Frio, RJ – Brasil; 2 Clínica Santa Helena – Cardiologia Cabo Frio RJ Brasil Clínica Santa Helena – Cardiologia, Cabo Frio, RJ – Brasil; 3 Fundação Técnico-Educacional Souza Marques Escola de Medicina Souza Marques Cabo Frio RJ Brasil Fundação Técnico-Educacional Souza Marques Escola de Medicina Souza Marques – Ensino e Graduação, Cabo Frio, RJ – Brasil; 4 Incordis Cabo Frio RJ Brasil Incordis, Cabo Frio, RJ – Brasil; 5 Complexo Hospitalar de Niterói Niterói RJ Brasil Complexo Hospitalar de Niterói, Niterói, RJ – Brasil; 6 Universidade Federal Fluminense Rio de Janeiro RJ Brasil Universidade Federal Fluminense, Rio de Janeiro, RJ – Brasil; 7 Universidade Federal do Rio de Janeiro Campus Macaé – Ensino e Graduação Macaé RJ Brasil Universidade Federal do Rio de Janeiro, Campus Macaé – Ensino e Graduação, Macaé, RJ – Brasil

**Keywords:** Estimulação do Nervo Vago, Insuficiência Cardíaca, Doenças do Sistema Nervoso Autônomo Parassimpático, Fração de Ejeção Reduzida

## Abstract

**Fundamento:**

O desequilíbrio do sistema nervoso autônomo (SNA) na insuficiência cardíaca (IC) cria um ciclo vicioso, o excesso de atividade simpática e a diminuição da atividade vagal contribuindo para a piora da IC. A estimulação elétrica transcutânea de baixa intensidade do ramo auricular do nervo vago (taVNS) é bem tolerada e abre novas possibilidades terapêuticas.

**Objetivos:**

Gerar hipótese da aplicabilidade e benefício da taVNS na IC através da comparação intergrupos de parâmetros ecocardiográficos, teste de caminhada de 6 min, variabilidade da frequência cardíaca pelo Holter (SDNN e rMSSD), questionário de qualidade de vida de Minnesota e classe funcional pela New York Heart Association.

**Métodos:**

Estudo clínico prospectivo, duplo cego, randomizado com metodologia sham, unicêntrico. Avaliados 43 pacientes e alocados em 2 grupos: o Grupo 1 recebeu taVNS (frequências 2/15 Hz) e Grupo 2 recebeu sham. Nas comparações, valores de p<0,05 foram considerados significativos.

**Resultados:**

Na fase pós-intervenção, observou-se que o Grupo 1 se manteve com melhor rMSSD (31 x 21; p = 0,046) e atingiu melhor SDNN (110 vs. 84, p = 0,033). Ao compararmos os parâmetros intragrupos, antes e após intervenção, observou-se que todos melhoraram significativamente no grupo 1 e não houve diferenças no grupo 2.

**Conclusão:**

A taVNS é uma intervenção segura, de fácil execução e que sugere provável benefício na IC pela melhora na variabilidade da frequência cardíaca, o que indica melhor equilíbrio autonômico. Novos estudos com maior número de pacientes são necessários para responder às questões levantadas por esse estudo.

## Introdução

A IC (insuficiência cardíaca) é considerada uma síndrome grave afetando, no mundo, mais de 23 milhões de pessoas.^1^ Sua mortalidade permanece elevada com sobrevida média de cinco anos após o diagnóstico de apenas 35% se não tratada.^2^ No Brasil, dados do registro BREATHE ( *Brazilian Registry of Acute Heart Failure* )^3^ mostraram a IC como principal causa de rehospitalizações, além de elevada taxa de mortalidade hospitalar.

Desequilíbrios do SNA (sistema nervoso autônomo) têm sido observados em diversas doenças^4^ e estão associados ao aumento do tônus simpático e diminuição do tônus parassimpático,^5^ como na IC,^6^ doenças inflamatórias intestinais e síndrome da dor crônica. A atividade simpática aumentada pode ser regulada por fármacos e a atividade parassimpática reduzida pode ser estimulada pelo treinamento físico, por exemplo.^7^

Recentemente, foi publicada metanálise^8^ mostrando que a estimulação invasiva do nervo vago melhorou a classe funcional pela NYHA ( *New York Heart Association* ), o teste de caminhada de 6 minutos (TC6min), a qualidade de vida pelo questionário de Minnesota (MLHFQ) e os níveis de NT-proBNP (fração N-terminal do peptídeo natriurético do tipo B) em pacientes com ICFER (IC com fração de ejeção reduzida).

A estimulação auricular do nervo vago (aVNS) é produzida por estimulação elétrica não invasiva do nervo vago na orelha,^9^ através de eletrodos (taVNS) ou pequenas agulhas (paVNS) colocadas na concha e/ou na parte inferior do tragus.

A regularização do equilíbrio autonômico mediada pela VNS diminui a atividade simpática e provoca a liberação de óxido nítrico^10^ o que, combinada com seus efeitos anti-inflamatórios, leva a uma melhora da oxigenação dos tecidos.^11^

Não há estudos atuais sobre taVNS na IC. No presente estudo buscamos analisar e gerar a hipótese da aplicabilidade e benefício da taVNS na ICFER através da comparação intergrupos do ecocardiograma, TC6min, variabilidade da frequência cardíaca pelo Holter (SDNN e rMSSD), MLHFQ^12^ e classe funcional (NYHA) aplicados antes de iniciar e ao finalizar as intervenções (taVNS e *Sham* ). Analisamos também a aplicabilidade e benefício da taVNS na ICFER através da comparação intragrupos dos dados supracitados.

## Métodos

Estudo clínico prospectivo, duplo cego, randomizado, com metodologia *sham* , sendo avaliados pacientes com IC e fração de ejeção < 50% em regime ambulatorial. Foram atendidos pacientes provenientes do ambulatório de IC da Secretaria de Saúde de Cabo Frio (CADHI-Centro de atendimento ao diabético, hipertenso e insuficiência cardíaca) e pacientes encaminhados por outros médicos para o ambulatório do Hospital Santa Izabel em Cabo Frio.

Ao estimularmos o nervo vago aferente em nível auricular modula-se o sistema nervoso autonômico cardíaco intrínseco para atingir o efeito cardioprotetor. Os pacientes foram estimulados a nível auricular até que percebessem um formigamento no local do estímulo, bem abaixo do limiar de dor, o que tornou exequível e confortável o procedimento.

Para evitar que o pesquisador tivesse conhecimento de quem recebeu taVNS ou *sham* foi escalada a enfermeira Rafaela dos Santos Cardoso Carneiro que, após treinamento e preparação adequados, realizou as intervenções e aplicou os testes. Coletaram-se dados através do acompanhamento dos pacientes e exames cardiológicos não invasivos como ecocardiograma e HOLTER ECG 24 h. A avaliação funcional foi abordada pelo TC6MIN, e a classe funcional-NYHA e o questionário de qualidade de vida Minnesota-MLHFQ foram também utilizados.

Utilizamos, em nosso estudo, o equipamento de eletroestimulação cutânea EL-30 (NKL Produtos Eletrônicos, Brusque, SC), com os seguintes parâmetros de estimulação: largura de pulso de 500 µs, intensidade abaixo do limiar doloroso, 5 segundos 2 Hz / 5 segundos 15 Hz. Estudos recentes mostraram que baixas frequências têm efeito maior na diminuição da atividade simpática,^13 , 14^ enquanto frequências na faixa de 10-25Hz produzem boa modulação parassimpática.^15^ Escolhemos o modo misto, utilizando tanto baixas (2 Hz) quanto médias frequências (15 Hz) de modo a obtermos ambos os benefícios autonômicos.

Foi usado ecocardiograma com alta qualidade de imagem e processamento, por meio da sonda Setorial Matricial XDclear, o Vivid S70N-GE.

O gravador digital de Holter Cardiolight-Cardios, com tecnologia digital de aquisição do sinal de 800 pontos por segundo com processamento em tempo real (DSP) foi utilizado em nosso estudo.

A intervenção (taVNS) ocorreu durante 30 minutos de segunda-feira a sexta-feira, totalizando 20 sessões. As avaliações e coleta de dados foram feitas antes de iniciar o estudo e após a última sessão de cada participante.

No período entre 03-02-2021 e 05-01-2022 foram inicialmente recrutados 52 pacientes, mas devido à pandemia de COVID-19 perdemos o seguimento de 9 pacientes. Logo, 43 pacientes concluíram o estudo, 22 pacientes no grupo taVNS e 21 no grupo *sham* .

A randomização foi realizada através de sorteio eletrônico e confecção de envelopes lacrados distribuídos de forma binária. À medida que os participantes eram recrutados um envelope era aberto: ao vir ‘0” (zero) recebia *sham* -simulado, ao vir “1” (um) recebia taVNS-intervenção.

Dessa forma, os pacientes foram alocados em 2 grupos:

*–* Grupo 1 (22 pacientes) recebeu a intervenção taVNS, com um eletrodo transcutâneo na concha superior (cimba) e o outro no lóbulo direito, nas frequências 2/15 Hz no período de 30 minutos. Estimulamos dessa forma o nervo vago na concha superior e o grande nervo auricular no lóbulo. Tais locais foram escolhidos baseados na inervação da orelha, facilidade técnica para colocação dos eletrodos e para uniformizar o tratamento.

**Figura 1:**
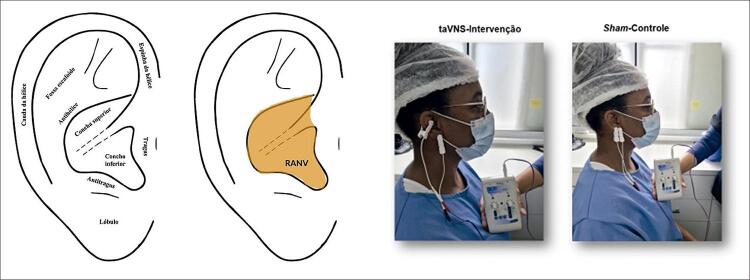
– Anatomia da orelha, mostrando a área de inervação pelo ramo auricular no nervo vago (RANV) e os locais de estimulação nos dois grupos, taVNS e sham. taVNS: estimulação transcutânea auricular do nervo vago.

*–* Grupo 2 (21 pacientes) recebeu a intervenção *sham* , com ambos os eletrodos transcutâneos no lóbulo direito nas frequências 2/15 Hz por período de 1 minuto, e depois desligado e mantido por 29 minutos. ( Figuras 1 e 2 )

**Figura 2:**
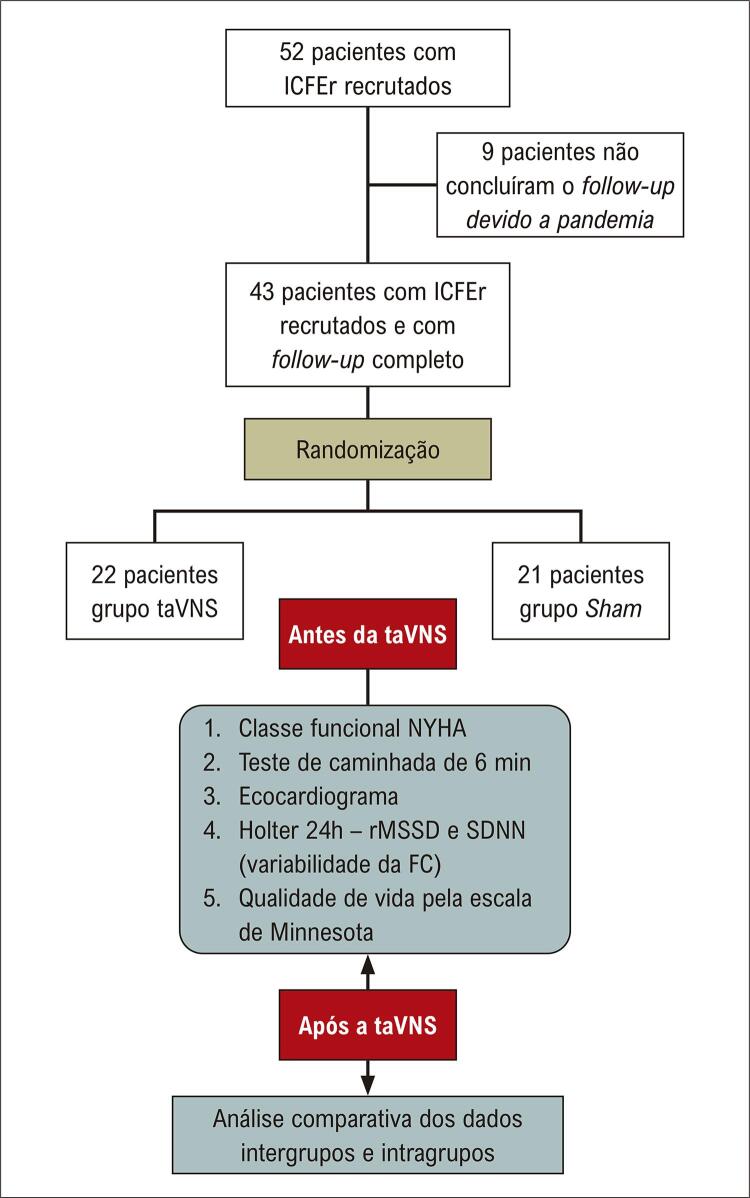
– Divisão dos grupos e fluxograma do estudo. taVNS: estimulação transcutânea auricular do nervo vago; Grupo sham: simulação. ICFER: insuficiência cardíaca com fração de ejeção reduzida; SDNN: desvio-padrão de todos os intervalos R-R normais gravados em um intervalo de tempo; rMSSD: raiz quadrada da média do quadrado das diferenças entre os intervalos R-R normais adjacentes, em um intervalo de tempo (milissegundos); FC: frequência cardíaca.

**Critérios de inclusão:**

- Pacientes ambulatoriais com IC compensada ou recuperada classes NYHA I-II-III e IV, recebendo terapia farmacológica ótima nos últimos 3 meses.
- Idade acima de 18 anos.
- FEVE menor que 50% documentada por ecocardiografia.

**Critérios de exclusão:**

- Pacientes com hospitalização por IC ou uso de terapia intravenosa para IC nos últimos 30 dias.
- Pacientes com insuficiência mitral grave ou estenose aórtica grave.
- Cirurgia cardíaca ou angioplastia ou AVC nos últimos 3 meses.
- Usuários de marcapasso.
- Pacientes com fração de ejeção ≥ 50%.

### Análise estatística

Com base em estudos anteriores,^16 - 18^ o presente estudo foi elaborado para detectar uma melhora de 30% nos escores de qualidade de vida, no teste de caminhada de 6 min e na variabilidade de FC no grupo taVNS (estimulação vagal) versus grupo *sham* . Um tamanho amostral de 40 pacientes (20 em cada grupo) forneceria pelo menos 80% de poder do teste para detectar essa diferença, em um nível alfa de significância de 0,05.

As variáveis contínuas foram apresentadas através de média ± desvio-padrão (DP) ou mediana (intervalo interquartil) conforme normalidade dos dados, e as variáveis categóricas foram apresentadas através de frequências absoluta e relativa.

Todas as variáveis contínuas foram testadas para normalidade pelo teste de Shapiro-Wilks.

As comparações nas características das variáveis contínuas entre os grupos foram realizadas por meio do teste *t* de Student não pareado (ou Mann-Whitney), e *t* de Student pareado para comparações intragrupos. O teste do qui-quadrado (ou exato de Fisher) foi usado para comparação entre as variáveis categóricas.

Valores de p < 0,05 foram considerados estatisticamente significativos e todos os testes foram bicaudais.

Todas as análises estatísticas foram realizadas utilizando-se o software *R Statistic* 3.5.1 ( *R Foundation for Statistical Computing* , Vienna, Austria).

### Recursos

- Humanos: A coleta de dados clínicos e a realização de exames cardiológicos foram feitos pelo investigador.
- Financeiros: Não houve recursos de terceiros, além de recursos próprios.

### Questões éticas

O pesquisador não teve conhecimento sobre a conduta clínica promovida pelos pacientes integrantes do estudo e provenientes do ambulatório de cardiologia, garantindo assim o tratamento ótimo para IC nos 2 grupos. Este estudo foi aprovado pelo CEP sob o parecer 4.486.173 em 29/12/2020 de conformidade com a resolução 466/2012 e cadastrado no ReBEC/World Health Organization, UTN: U111112552081, e na Plataforma Brasil: 38606820.6.0000.5243.

## Resultados

As características clínicas basais foram similares na maioria dos parâmetros nos 2 grupos ( Tabela 1 ). No entanto, na fase pré-intervenção o Grupo 1 (taVNS) apresentou idade superior (p= 0,037) e maior rMSSD (p= 0,018).

**Tabela 1:** – Características clínicas basais – variáveis categóricas

	Pacientes (n=43)	Grupos		Valor de p

		taVNS (n=22)	Controle (n=21)	
Sexo masculino, %	79,1	72,7	85,7	0,457
Classe NYHA, n (%)				0,186
I	12 (27,9)	3 (13,6)	9 (42,9)	
II	17 (39,5)	10 (45,5)	7 (33,3)	
III	11 (25,6)	7 (31,8)	4 (19,0)	
IV	3 (7,0)	2 (9,1)	1 (4,8)	
Hipertensão arterial, n (%)	36 (86,7)	19 (86,4)	17 (81,0)	0,698
Dislipidemia, n (%)	19 (44,2)	12 (54,5)	7 (33,3)	0,223
HF de DAC, n (%)	23 (53,5)	13 (59,1)	10 (47,6)	0,547
Diabetes, n (%)	17 (39,5)	10 (45,5)	7 (33,3)	0,536
Tabagismo, n (%)	2 (4,7))	1 (95,5)	1 (95,2)	1
Obesidade, n (%)	5 (11,6)	4 (18,2)	1 (4,8)	0,345
Etilismo/drogas, n (%)	2 (4,7)	2 (9,1)	0	0,488
DAP, n (%)	12 (27,9)	8 (36,4)	4 (19,0)	0,310
IAM prévio, n (%)	21 (48,8)	11 (50)	10 (47,6)	1
ICP prévia, n (%)	10 (23,3)	6 (27,3)	4 (19,0)	0,721
RM prévia, n (%)	6 (14)	3 (13,6)	3 (14,3)	1
Etiologia da IC, n (%)				1
Hipertensiva	15 (34,9)	8 (36,4)	7 (33,3)	
Indeterminada	14 (32,6)	7 (31,8)	7 (33,3)	
Isquêmica	14 (32,6)	7 (31,8)	7 (33,3)	
Ritmo sinusal, n (%)	38 (88,4)	20 (90,9)	18 (85,7)	0,664
Internação prévia, n (%)	22 (51,2)	10 (45,5)	12 (57,1)	0,547

*NYHA: New York Heart Association; HF de DAC: história familiar de doença arterial coronária; DAP: doença arterial periférica; IAM: infarto agudo do miocárdio; ICP: intervenção coronária percutânea; RM: cirurgia de revascularização do miocárdio; IC: insuficiência cardíaca. Testes estatísticos realizados: teste do qui-quadrado ou teste exato de Fisher. Valores de p < 0,05 foram considerados significativos.*

O Grupo 2 ( *sham)* na fase pré-intervenção apresentou melhor qualidade de vida (p= 0,013) e tendência a melhor desempenho no TC6M (292 vs. 365, p= 0,09) como se pode observar na Tabela 2 .

**Tabela 2:** – Características clínicas basais – variáveis numéricas

	Pacientes (n=43)	Grupos		Valor de p

		taVNS (n=22)	Controle (n=21)	
Idade	60,7 ± 12,7	64,6 ± 11,2	56,6 ± 13,1	0,037
Peso	82,4 ± 17,5	84,5 ± 18,1	80,2 ± 17,1	0,436
Tempo diagnóstico IC (anos)	5 (4)	5 (3,8)	3 (4)	0,742
FEVE	0,35 ± 0,1	0,34 ± 0,1	0,36 ± 0,1	0,441
VSFVE (mm)	51,9 ± 11,1	54,0 ± 12,1	49,8 ± 9,6	0,206
VDFVE (mm)	63 (9)	64 (8,8)	61 (9)	0,201
AE (mm)	44 (6,5)	45 (7,5)	44 (6)	0,193
TC6M (min)	328,9 ± 137,1	292,2 ± 143,2	365,6 ± 123,3	0,090
MLHFQ	57 ± 17,6	63,5 ± 16,0	50,4 ± 17,1	0,013
SDNN (ms)	96 (53)	103 (74,2)	94 (37)	0,148
rMSSD (ms)	29 (53,5)	37 (87,2)	28 (16)	0,018

*Variáveis contínuas representadas por média ± desvio-padrão ou mediana (intervalo interquartil); IC: insuficiência cardíaca; FEVE: fração de ejeção do ventrículo esquerdo; VSFVE: volume sistólico final do ventrículo esquerdo; VDFVE: volume diastólico final do ventrículo esquerdo; AE: átrio esquerdo; MLHQ: Minnesota Living with Heart Failure Questionnaire; SDNN: desvio-padrão de todos os intervalos R-R normais gravados em um intervalo de tempo; rMSSD: raiz quadrada da média do quadrado das diferenças entre os intervalos R-R normais adjacentes, em um intervalo de tempo (milissegundos). Testes estatísticos utilizados: teste t de Student não pareado para variáveis simétricas (exibidas como média ± DP) e teste de Mann-Whitney para variáveis assimétricas [exibidas como mediana (intervalo interquartil)]. Valores de p < 0,05 foram considerados significativos.*

Na fase pós-intervenção observou-se que o Grupo 1 se manteve com melhor rMSSD (31 vs. 21; p = 0,046) e atingiu melhor SDNN (110 vs. 84, p = 0,033) ( Tabela 3 ). Nos demais parâmetros não houve diferenças entre os grupos.

**Tabela 3:** – Diferença entre os grupos após 4 semanas

	Pacientes (n=43)	Grupos		Valor de p

		TaVNS (n=22)	Controle (n=21)	
Mediana da classe da NYHA	1 (1)	2 (1)	1 (1)	0,232
FEVE-Simpson (%)	0,37 ± 0,1	0,37 ± 0,1	0,36 ± 0,05	0,686
VSFVE (mm)	51,3 ± 7,8	53,1 ± 8,8	49,4 ± 6,4	0,124
VDFVE (mm)	65 (10)	66,5 (8,2)	64 (11)	0,237
AE (mm)	42,6 (6,5)	41 (5)	41 (6)	0,129
TC6M (min)	378,9 ± 138,8	353 ± 119,7	405,9 ± 154,6	0,219
MLHFQ	48,9 ± 13,4	48,6 ± 11,9	49,1 ± 15,2	0,913
SDNN (ms)	99 (62,5)	110 (64)	84 (44)	0,033
rMSSD (ms)	26 (31)	31 (77,2)	21 (20)	0,046

*Variáveis contínuas representadas por média ± desvio-padrão ou mediana (intervalo interquartil); IC: insuficiência cardíaca; FEVE: fração de ejeção do ventrículo esquerdo; VSFVE: volume sistólico final do ventrículo esquerdo; VDFVE: volume diastólico final do ventrículo esquerdo; AE: átrio esquerdo; MLHQ: Minnesota Living with Heart Failure Questionnaire; SDNN: desvio-padrão de todos os intervalos R-R normais gravados em um intervalo de tempo; rMSSD: raiz quadrada da média do quadrado das diferenças entre os intervalos R-R normais adjacentes, em um intervalo de tempo (milissegundos). Testes estatísticos utilizados: teste t de Student não pareado para variáveis simétricas (exibidas como média ± DP) e teste de Mann-Whitney para variáveis assimétricas [exibidas como mediana (intervalo interquartil)]. Valores de p < 0,05 foram considerados significativos.*

**Figura 3:**
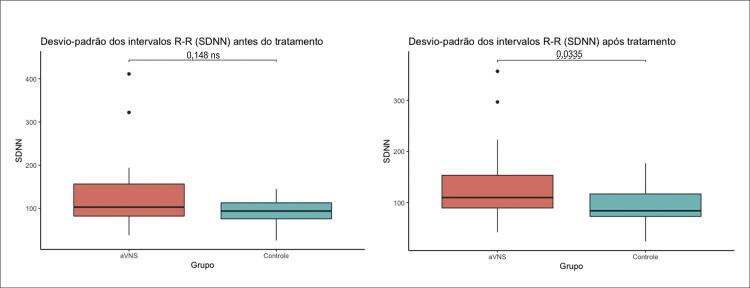

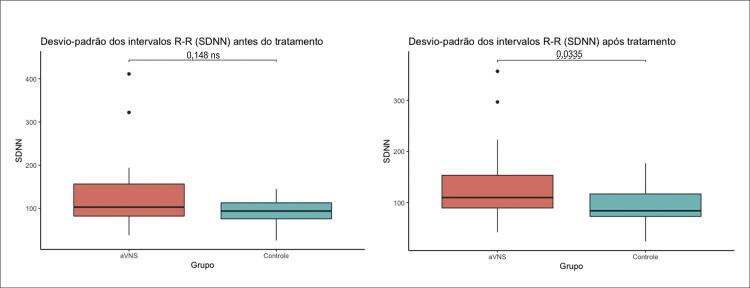
– Desvio-Padrão do R-R (SDNN) antes e após o tratamento. Teste estatístico realizado: t de Student não pareado. Valores de p < 0,05 foram considerados significativos. SDNN: desvio-padrão de todos os intervalos R-R normais gravados em um intervalo de tempo; aVNS: estimulação auricular do nervo vago.

Nota-se que o SDNN nos dois grupos antes da taVNS apresentava níveis semelhantes, mas analisando a Figura 3 podemos observar que após a intervenção o grupo 1 atingiu melhor SDNN e não foi observado o mesmo benefício no grupo 2.

Ao comparar os parâmetros antes e após a intervenção na análise intragrupos, constatamos que muitos melhoraram significativamente no Grupo 1 e não houve diferença no Grupo 2.

**Figura 4:**
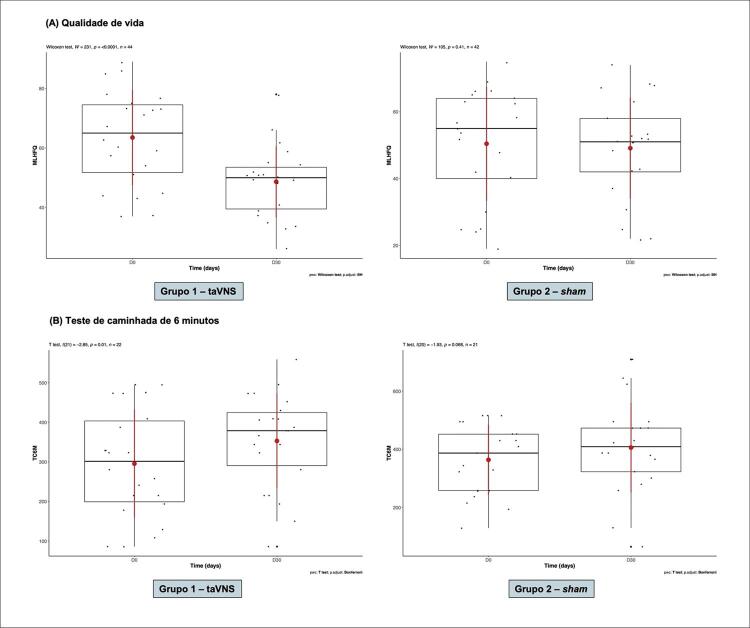
– Análise da qualidade de vida pelo questionário de Minnesota-MLHFQ (A) e do teste de caminhada dos 6 minutos (B) nos grupos 1 (taVNS) e 2 (sham) antes do tratamento e após 30 dias. Nota-se, em ambos os casos, melhora desses parâmetros no grupo taVNS (p<0,05) e nenhuma melhora no grupo sham. Testes estatísticos realizados: Wilcoxon-Mann-Whitney para qualidade de vida e t de Student pareado para o teste de caminhada de 6 minutos. Valores de p < 0,05 foram considerados significativos.

Houve benefício no Grupo 1 após taVNS quanto à qualidade de vida, enquanto não houve o mesmo benefício no grupo controle após 30 dias de estimulação ( Figura 4 ). Da mesma maneira, foi observada superioridade do Grupo 1 no TC6M quando comparamos antes e após a intervenção, fato que não ocorreu no Grupo 2 ( Figura 4 ).

Não observamos nenhuma intercorrência ou abandono do tratamento por eventos adversos em nosso estudo.

O resumo do design e dos achados do estudo pode ser observado na ( Figura Central ).

**Figura Central:**
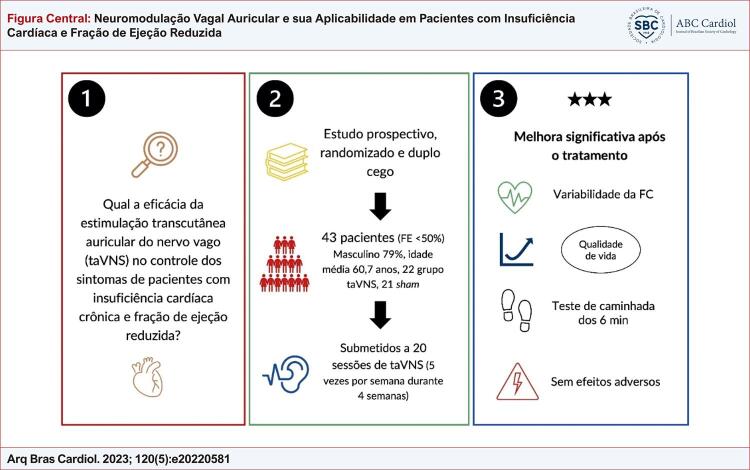
: Neuromodulação Vagal Auricular e sua Aplicabilidade em Pacientes com Insuficiência Cardíaca e Fração de Ejeção Reduzida

Análise de dados e resultados. taVNS: estimulação transcutânea auricular do nervo vago (intervenção); FE: fração de ejeção; sham: simulação (controle); FC: frequência cardíaca; 6 min: 6 minutos.

## Discussão

Este estudo mostrou que, em pacientes com ICFEr, quando comparamos o estímulo com taVNS vs. *sham* , houve melhora do índice de variabilidade de frequência cardíaca no grupo intervenção, não havendo benefícios nos demais parâmetros. Por outro lado, quando se comparou as variáveis intragrupos, observou-se melhora no TC6M e MLHFQ após a taVNS, enquanto não se modificaram no grupo controle.

Sabemos desde 1998 com o estudo de Nolan et al.^19 , 20^ que a redução da variabilidade cardíaca é um preditor independente no aumento na morte súbita na IC e mesmo na população geral.^21^ Podemos sugerir que a taVNS, trazendo um aumento na variabilidade da FC, possa estar associada à redução de morte súbita^21 - 23^ por interferir indiretamente na redução da cascata inflamatória da IC, com menor carga arrítmica, através de um melhor equilíbrio neurohumoral.

De acordo com os estudos HOPE4 HF^16^ e BEAT HF^24^ o uso de terapia de ativação do barorreflexo foi segura e conferiu benefício na IC. O presente estudo demonstrou a mesma segurança, facilidade de execução e menores efeitos colaterais, além de mostrar benefício na variabilidade da FC e sugerir melhoras no TC6M e na qualidade de vida. Com a melhora da capacidade funcional, foi perceptível em todos os pacientes o desejo de persistir no tratamento mesmo durante um período de pandemia e riscos.

Frangos et al. em 2015^25^ mostraram o benefício e a facilidade da execução da taVNS em humanos de forma não invasiva e foi possível confirmar neste estudo a mesma facilidade de execução.

Zannad et al., no estudo NECTAR HF,^17^ usando VNS, falharam no objetivo primário quanto à melhoria das medidas ecocardiográficas após VNS, mas demonstraram melhoria na qualidade de vida, achado este que foi possível demonstrar em nosso estudo sem a necessidade de intervenção invasiva.

A melhora na qualidade de vida acarretou uma melhor adesão ao tratamento, no estilo de vida, uma perceptível satisfação do paciente e um engajamento maior ao perceber resultados palpáveis e um novo foco sobre seu posicionamento quanto à IC e suas expectativas.

Gold et al., no estudo INOVATE HF,^18^ envolvendo 85 centros, não demonstraram redução da mortalidade ao utilizar VNS, mas sim um benefício no teste de caminhada de 6 minutos, o que se alinha com nossos achados, com a vantagem que utilizamos a via auricular do nervo vago.

A classe funcional da NYHA e a qualidade de vida melhoraram após VNS em vários estudos.^18 , 26^ Esses efeitos positivos demonstraram que a maioria dos pacientes se tornaram menos sintomáticos e mais capazes para as atividades do dia-a-dia após o tratamento com VNS. O teste de caminhada de seis minutos foi realizado em cinco estudos relativamente recentes, com aumento significativo da distância percorrida em pacientes tratados por VNS.^27 , 28^ Esses achados se alinham com a melhora no TC6M e na qualidade de vida observados neste estudo no grupo taVNS, apontando que esses pacientes se tornaram fisicamente mais aptos após a estimulação vagal. Por outro lado, o presente estudo não foi capaz de demonstrar melhora na classe funcional NYHA, provavelmente porque a maior parte dos pacientes já estavam em classes I ou II desde o início.

No estudo ANTHEM HF, Premchand et al.^29^ demonstraram que a VNS no lado esquerdo ou direito não apresentou diferença nos resultados e foi seguro. Neste estudo, optou-se por manter o estímulo no ouvido externo direito por simples convenção.

Em 2015 e novamente em 2020, no estudo TREAT AF, Stavrakis et al.^30 , 31^ demonstraram que taVNS suprimiu e reduziu a carga de fibrilação atrial em pacientes sem IC, além de reduzir os níveis de citocinas pró-inflamatórias. Recentemente, o mesmo grupo demonstrou, num estudo piloto, que a taVNS reduziu os níveis de fator de necrose tumoral alfa e melhorou a qualidade de vida em pacientes com IC com fração de ejeção preservada.^32^ Nosso estudo vem gerar a hipótese que a taVNS possa vir também a ser benéfica em pacientes com IC com fração de ejeção reduzida, pois observou-se melhora na variabilidade de frequência cardíaca no grupo taVNS.

Kaniusas et al.^33 , 34^ demonstraram de forma sistemática os efeitos benéficos e anti-inflamatórios da taVNS, não apenas pelos mecanismos clássicos expostos em seus estudos, mas também por outros ainda não bem compreendidos. Este estudo vem gerar uma hipótese ao demonstrar que, ao modularmos o excesso de atividade simpática e estimularmos a atividade parassimpática, obtivemos resultados promissores na insuficiência cardíaca.

Em recente publicação, Sant’Anna et al.^8^ realizaram metanálise sobre estudos clínicos randomizados comparando VNS invasiva + tratamento medicamentoso vs. tratamento medicamentoso na IC, e observaram que em pacientes com ICFEr o uso de VNS foi associado à melhora na classe funcional NYHA, qualidade de vida, TC6M e redução dos níveis de NT-proBNP. Neste estudo foi possível observar melhora na variabilidade da frequência cardíaca, qualidade de vida e TC6M, com menos efeitos adversos que os estudos invasivos e que usaram dispositivos implantáveis.

### Limitações

Este estudo apresentou algumas limitações:

1. O Grupo 1 teve na fase pré-intervenção, idade superior, pior qualidade de vida e maior rMSSD que o Grupo 2, o que pode prejudicar as análises após a intervenção. Atribuímos tal achado ao pequeno tamanho de amostra, mas os resultados mostraram que tais discrepâncias não influíram nos achados finais;
2. A pandemia de COVID-19 foi um obstáculo para realização desse estudo. Ressalta-se a preocupação dos pacientes por serem cardiopatas e do risco de contágio. Tais obstáculos foram contornados mudando o ambiente e informando que seriam fornecidas medidas protetivas, embora tal fato não tenha afetado a análise dos dados;
3. Outra limitação trazida pela pandemia foi a crise econômica dificultando a mobilização para realizar o tratamento e exames seriados. Fornecemos passagem, ajuda de custo alimentar e o fundamental esclarecimento sobre a importância do tratamento;
4. Não dosamos biomarcadores neste estudo, no entanto, até à época do recrutamento não dispúnhamos de laboratório disponível em nossa região. No entanto, a ideia original era gerar hipótese para tratamento ambulatorial, o que foi feito;
5. Uma importante limitação decorre do curto prazo do estudo. A maioria dos estudos de estimulação vagal demonstraram um resultado mais perceptível após um período maior de estimulação, enquanto o período de tratamento deste estudo foi de apenas 1 mês. Como ainda assim os resultados foram promissores, espera-se que novos estudos venham em breve esclarecer o tempo mínimo e o ideal para se obter um efeito razoável da modulação vagal na IC;
6. Outro fator limitante foi a classe funcional NYHA dos pacientes, a maioria foi classe I (27,9%) ou II (39,5%), e com isso o objetivo de avaliar a melhora de classe funcional nesses pacientes perdeu um pouco o sentido. Novos estudos envolvendo uso de taVNS no tratamento da IC devem excluir a classe I da NYHA, uma vez que nesses pacientes já se atingiu o benefício clínico almejado.

## Conclusão

A taVNS é uma intervenção segura, de fácil execução e pode conferir benefício na IC pela melhora nos parâmetros de variabilidade de frequência cardíaca (SDNN), o que indica melhor equilíbrio autonômico. Mostrou-se também, na comparação intragrupos antes e após o tratamento, melhora na qualidade de vida e no teste de caminhada de 6 minutos no grupo taVNS.

A partir desses resultados, pode-se aventar a hipótese de ampliação da indicação da neuromodulação vagal auricular em pacientes com IC, embora novos estudos com maior número de pacientes sejam necessários para responder às questões levantadas pelo presente estudo.
