# Supplementary material for: Neuromodulação Vagal Auricular e sua Aplicabilidade em Pacientes com Insuficiência Cardíaca e Fração de Ejeção Reduzida
Source: Arq Bras Cardiol. 2023 May 2;120(5):e20220581. [Article in Portuguese] doi: 10.36660/abc.20220581 (PMC10263391; doi:10.36660/abc.20220581)
Supplement: Translation

# Translation: Auricular Vagal Neuromodulation and its Application in Patients with Heart Failure and Reduced Ejection Fraction

**Authors:** Sergio Menezes Couceiro, Lucas Bonacossa Sant’Anna, Mariana Bonacossa Sant’Anna, Renata S. Matos Menezes, Evandro Tinoco Mesquita, Fernando Mendes Sant’Anna

**Affiliations:** 1 Brazil Universidade Federal Fluminense, Cabo Frio, RJ – Brazil; 2 Brazil Clínica Santa Helena – Cardiologia, Cabo Frio, RJ – Brazil; 3 Brazil Fundação Técnico-Educacional Souza Marques Escola de Medicina Souza Marques – Ensino e Graduação, Cabo Frio, RJ – Brazil; 4 Brazil Incordis, Cabo Frio, RJ – Brazil; 5 Brazil Complexo Hospitalar de Niterói, Niterói, RJ – Brazil; 6 Brazil Universidade Federal Fluminense, Rio de Janeiro, RJ – Brazil; 7 Brazil Universidade Federal do Rio de Janeiro, Campus Macaé – Ensino e Graduação, Macaé, RJ – Brazil

**Keywords:** Vagus Nerve Stimulation, Heart Failure, Parasympathetic Nervous System Diseases, Reduced Ejection Fraction

## Abstract

**Background:**

The autonomic nervous system (ANS) imbalance in heart failure (HF) creates a vicious cycle, excess sympathetic activity, and decreased vagal activity contributing to the worsening of HF. Low-intensity transcutaneous electrical stimulation of the auricular branch of the vagus nerve (taVNS) is well tolerated and opens new therapeutic possibilities.

**Objectives:**

To hypothesize the applicability and benefit of taVNS in HF through intergroup comparison of echocardiography parameters, 6-minute walk test, Holter heart rate variability (SDNN and rMSSD), Minnesota quality of life questionnaire, and functional class by the New York Heart Association. In comparisons, p values <0.05 were considered significant.

**Methods:**

Prospective, double-blind, randomized clinical study with sham methodology, unicentric. Forty-three patients were evaluated and divided into 2 groups: Group 1 received taVNS (frequencies 2/15 Hz), and Group 2 received sham. In comparisons, p values <0.05 were considered significant.

**Results:**

In the post-intervention phase, it was observed that Group 1 had better rMSSD (31 x 21; p = 0.046) and achieved better SDNN (110 vs. 84, p = 0.033). When comparing intragroup parameters before and after the intervention, it was observed that all of them improved significantly in group 1, and there were no differences in group 2.

**Conclusion:**

taVNS is a safe to perform and easy intervention and suggests a probable benefit in HF by improving heart rate variability, which indicates better autonomic balance. New studies with more patients are needed to answer the questions raised by this study.

## Introduction

Heart failure (HF) is considered a serious syndrome affecting more than 23 million people worldwide.^1^Its mortality remains high, with average five-year survival after diagnosis of only 35% if untreated.^2^In Brazil, data from the BREATHE registry (Brazilian Registry of Acute Heart Failure)^3^showed HF as the main cause of rehospitalizations and a high hospital mortality rate.

ANS (autonomic nervous system) imbalances have been observed in several diseases^4^and are associated with increased sympathetic tonus and decreased parasympathetic tonus,^5^such as in HF,^6^inflammatory bowel diseases, and chronic pain syndrome. Drugs can regulate the increased sympathetic activity, and the reduced parasympathetic activity can be stimulated by physical training, for example.^7^

Recently, a meta-analysis^8^was published showing that invasive stimulation of the vagus nerve improved the functional class by the New York Heart Association(NYHA), the 6-minute walk test (6MWT), the quality of life by the Minnesota questionnaire (MLHFQ) and the NT-proBNP levels (N-terminal fraction of pro-B-type natriuretic peptide) in patients with HFrER (heart failure with reduced ejection fraction).

Auricular vagus nerve stimulation (aVNS) is produced by non-invasive electrical stimulation of the vagus nerve in the ear^9^through electrodes (taVNS) or small needles (paVNS) placed in the concha and/or lower part of the tragus.

The regulation of autonomic balance mediated by VNS decreases sympathetic activity and causes the release of nitric oxide,^10^which, combined with its anti-inflammatory effects, leads to improved tissue oxygenation.^11^

There are no current studies on taVNS in HF. In the present study, we sought to analyze and hypothesize the applicability and benefit of taVNS in HFrER by comparing intergroup echocardiographic parameters, 6MWT, Holter heart rate variability (SDNN and rMSSD), MLHFQ^12^and functional class (NYHA) applied before starting and at the end of the interventions (taVNS and *sham* ). We also analyzed the applicability and benefit of taVNS in HFrER by intragroup comparison of the abovementioned data.

## Methods

A prospective, double-blind, randomized clinical study, with sham methodology, evaluating patients with HF and ejection fraction < 50% on an outpatient basis. Patients from the HF outpatient clinic of the Cabo Frio Health Secretariat (CADHI-Center for diabetic, hypertensive, and heart failure care) and patients referred by other physicians to the outpatient clinic of Hospital Santa Izabel in Cabo Frio were treated.

When we stimulate the afferent vagus nerve at the auricular level, the intrinsic cardiac autonomic nervous system is modulated to achieve the cardioprotective effect. Patients were stimulated at the auricular level until they felt tingling at the stimulus site, well below the pain threshold, which made the procedure feasible and comfortable.

To prevent the researcher from knowing who received taVNS or sham, nurse Rafaela dos Santos Cardoso Carneiro was chosen, who, after adequate training and preparation, carried out the interventions and applied the tests. Data were collected through patient follow-up and non-invasive cardiological tests such as echocardiography and 24-hour HOLTER ECG. The 6MWT addressed functional assessment, and the NYHA functional class and the Minnesota quality of life questionnaire (MLHFQ) were also used.

Our study used the EL-30 electrical stimulation equipment (NKL Electronic Products, Brusque, SC) with the following stimulation parameters: pulse width of 500 µs, intensity below the painful threshold, 5 seconds 2 Hz / 5 seconds 15 Hz. Recent studies have shown that low frequencies have a greater effect on decreasing sympathetic activity,^13 , 14^while frequencies in the 10-25Hz range produce good parasympathetic modulation.^15^We chose the mixed mode, using both low (2 Hz) and medium frequencies (15 Hz) to obtain both autonomic benefits.

A high image and processing quality echocardiogram was used, the Vivid S70N-GE, with the XDclear Matrix Sector probe.

The Holter Cardiolight-Cardios digital recorder, with digital signal acquisition technology at 800 points per second with real-time processing (DSP), was employed in our study.

The intervention (taVNS) took 30 minutes from Monday to Friday, totaling 20 sessions. Assessments and data collection were performed before starting the study and after the last session of each participant.

From 2021-02-03 to 2022-01-05, 52 patients were initially recruited, but due to the COVID-19 pandemic, we lost the follow-up of 9 patients. Therefore, 43 patients completed the study, 22 in the taVNS group and 21 in the *sham* group.

Randomization was carried out through an electronic raffle and the creation of sealed envelopes distributed in a binary way. As participants were recruited, an envelope was opened: when ‘0’ (zero) came up, they received the *sham* intervention; when ‘1’ (one) came up, they received taVNS.

Thus, patients were allocated into 2 groups:

- Group 1 (22 patients) received the taVNS intervention, with a transcutaneous electrode on the superior concha (cimba) and the other on the right lobe, at frequencies 2/15 Hz over 30 minutes. This way, we stimulate the superior concha’s vagus nerve and the lobe’s great auricular nerve. Such sites were chosen based on the innervation of the ear, the technical facility for placing the electrodes and standardizing the treatment.
- Group 2 (21 patients) received the *sham* intervention, with both transcutaneous electrodes on the right lobe at frequencies 2/15 Hz for 1 minute, then turned off and maintained for 29 minutes. ( Figures 1 e 2 )
  **Figure 1:** – Anatomy of the ear, showing the area of innervation by the auricular branch in the vagus nerve (ABVN) and the stimulation sites in both groups, taVNS, and sham. taVNS: transcutaneous auricular vagus nerve stimulation; Sham: simulation.
  **Figure 2:** – Study flowchart. taVNS: transcutaneous auricular vagus nerve stimulation; Sham group: simulation; HFrEF: heart failure with reduced ejection fraction; SDNN: standard deviation of all normal R-R intervals recorded in a time interval; rMSSD: square root of the mean squared differences between adjacent normal R-R intervals over a time interval (milliseconds).

**Inclusion criteria:**

- Outpatients with compensated or recovered HF NYHA classes I-IV, receiving optimal pharmacological therapy in the last 3 months.
- Age over 18 years old.
- LVEF (left ventricle ejection fraction) less than 50% documented by echocardiography.

**Exclusion criteria:**

- Patients hospitalized for HF or using intravenous therapy for HF in the last 30 days.
- Patients with severe mitral regurgitation or severe aortic stenosis.
- Heart surgery or angioplasty, or stroke within the last 3 months.
- Pacemaker users.
- Patients with an LVEF ≥ 50%.

### Statistical analysis

Based on previous studies,^16 - 18^the present study was designed to detect a 30% improvement in quality of life scores, 6-min walk test, and HR variability in the taVNS group versus the *sham* group. A sample size of 40 patients (20 in each group) would provide at least 80% test power to detect this difference at an alpha significance level of 0.05.

Continuous variables were presented as mean ± standard deviation (SD) or median (interquartile range) according to data normality, and categorical variables were presented as absolute and relative frequencies. All continuous variables were tested for normality using the Shapiro-Wilks test.

Comparisons in the characteristics of continuous variables between groups were performed using the unpaired Student’s t-test (or Mann-Whitney) and paired Student’s t-test for intragroup comparisons. The chi-square (or Fisher’s exact) test was used to compare categorical variables.

P values < 0.05 were considered statistically significant, and all tests were two-tailed. All statistical analyzes were performed using the software *R Statistic* 3.5.1 ( *R Foundation for Statistical Computing* , Vienna, Austria).

### Resources

- Humans: the main investigator performed the collection of clinical data and the performance of cardiological examinations.
- Financial: there were no resources from third parties besides our own resources.

### Ethical issues

The researcher was unaware of the clinical conduct promoted by the patients participating in the study and coming from the cardiology outpatient clinic, thus guaranteeing the optimal treatment for HF in the 2 groups. This study was approved by the Ethical Committee under opinion 4,486,173 on 12/29/2020 following resolution 466/2012 and registered at ReBEC (Brazilian Registry of Clinical Trials), UTN: U111112552081, and at Plataforma Brasil: 38606820.6.0000.5243.

## Results

Baseline clinical characteristics were similar in most parameters in the 2 groups ( Table 1 ). However, in the pre-intervention phase, Group 1 (taVNS) were older (p= 0.037) and showed higher rMSSD (p= 0.018).

**Table 1:** – Baseline clinical characteristics – categorical variables

	Patients (n=43)	Groups		p-value
		taVNS (n=22)	*Sham* (n=21)	
Male, %	79.1	72.7	85.7	0.457
NYHA class, n (%)				0.186
I	12 (27.9)	3 (13.6)	9 (42.9)	
II	17 (39.5)	10 (45.5)	7 (33.3)	
III	11 (25.6)	7 (31.8)	4 (19.0)	
IV	3 (7.0)	2 (9.1)	1 (4.8)	
Arterial hypertension, n (%)	36 (86.7)	19 (86.4)	17 (81.0)	0.698
Dyslipidemia, n (%)	19 (44.2)	12 (54.5)	7 (33.3)	0.223
FH of CAD, n (%)	23 (53.5)	13 (59.1)	10 (47.6)	0.547
Diabetes, n (%)	17 (39.5)	10 (45.5)	7 (33.3)	0.536
Smoke, n (%)	2 (4.7))	1 (95.5)	1 (95.2)	1
Obesity, n (%)	5 (11.6)	4 (18.2)	1 (4.8)	0.345
Alcoholism/drugs, n (%)	2 (4.7)	2 (9.1)	0	0.488
PVD, n (%)	12 (27.9)	8 (36.4)	4 (19.0)	0.310
Previous AMI, n (%)	21 (48.8)	11 (50)	10 (47.6)	1
Previous PCI, n (%)	10 (23.3)	6 (27.3)	4 (19.0)	0.721
CABG, n (%)	6 (14)	3 (13.6)	3 (14.3)	1
HF etiology, n (%)				1
Hypertensive	15 (34.9)	8 (36.4)	7 (33.3)	
Idiopathic	14 (32.6)	7 (31.8)	7 (33.3)	
Ischemic	14 (32.6)	7 (31.8)	7 (33.3)	
Sinus rhythm, n (%)	38 (88.4)	20 (90.9)	18 (85.7)	0.664
Previous hospitalization, n (%)	22 (51.2)	10 (45.5)	12 (57.1)	0.547

*NYHA: New York Heart Association; FH of CAD: familial history of coronary artery disease; PVD: peripheral vascular disease; AMI: acute myocardial infarction; PCI: percutaneous coronary intervention; CABG: coronary artery bypass graft; HR: heart failure. Statistical tests performed: chi-square test or Fisher’s exact test. P values < 0.05 were considered significant.*

Group 2 ( *sham* ) in the pre-intervention phase had a better quality of life (p= 0.013) and a tendency to better performance in the 6MWT (292 vs. 365, p= 0.09), as displayed in Table 2 .

**Table 2:** – Baseline clinical characteristics – numerical variables

	Patients (n=43)	Groups		p-value
		taVNS (n=22)	*Sham* (n=21)	
Age	60.7 ± 12.7	64.6 ± 11.2	56.6 ± 13.1	0.037
Weight	82.4 ± 17.5	84.5 ± 18.1	80.2 ± 17.1	0.436
HF diagnostic time (years)	5 (4)	5 (3.8)	3 (4)	0.742
LVEF	0.35 ± 0.1	0.34 ± 0.1	0.36 ± 0.1	0.441
LVESV (mm)	51.9 ± 11.1	54.0 ± 12.1	49.8 ± 9.6	0.206*
LVEDV (mm)	63 (9)	64 (8.8)	61 (9)	0.201
LA (mm)	44 (6.5)	45 (7.5)	44 (6)	0.193
6MWT (min)	328.9 ± 137.1	292.2 ± 143.2	365.6 ± 123.3	0.090
MLHFQ	57 ± 17.6	63.5 ± 16.0	50.4 ± 17.1	0.013
SDNN (ms)	96 (53)	103 (74.2)	94 (37)	0.148
rMSSD (ms)	29 (53.5)	37 (87.2)	28 (16)	0.018

*Continuous variables represented by mean ± standard deviation or median (interquartile range); HF: heart failure; LVEF: left ventricular ejection fraction; LVESV: left ventricular end-systolic volume; LVEDV: left ventricular end-diastolic volume; LA: left atrium; 6MWT: six-minute walking test; MLHQ: Minnesota Living with Heart Failure Questionnaire; SDNN: standard deviation of all normal R-R intervals recorded in a time interval; rMSSD: square root of the mean squared differences between adjacent normal R-R intervals over a time interval (milliseconds). Statistical tests used: unpaired Student’s t-test for symmetrical variables (displayed as mean ± SD) and Mann-Whitney test for asymmetrical variables [displayed as median (interquartile range)]. P values < 0.05 were considered significant.*

In the post-intervention phase, it was observed that Group 1 maintained a better rMSSD (31 vs. 21; p = 0.046) and achieved a better SDNN (110 vs. 84, p = 0.033) ( Table 3 ). There were no differences between the groups for the other parameters.

**Table 3:** – Differences between groups after 4 weeks

	Patients (n=43)	Groups		p-value
		taVNS (n=22)	*Sham* (n=21)	
NYHA class median	1 (1)	2 (1)	1 (1)	0.232
LVEF-Simpson (%)	0.37 ± 0.1	0.37 ± 0.1	0.36 ± 0.05	0.686
LVESV (mm)	51.3 ± 7.8	53.1 ± 8.8	49.4 ± 6.4	0.124
LVEDV (mm)	65 (10)	66.5 (8.2)	64 (11)	0.237
LA (mm)	42.6 (6.5)	41 (5)	41 (6)	0.129
6MWT (min)	378.9 ± 138.8	353 ± 119.7	405.9 ± 154.6	0.219
MLHFQ	48.9 ± 13.4	48.6 ± 11.9	49.1 ± 15.2	0.913
SDNN (ms)	99 (62.5)	110 (64)	84 (44)	0.033
rMSSD (ms)	26 (31)	31 (77.2)	21 (20)	0.046

*Continuous variables are represented by mean ± standard deviation or median (interquartile range). NYHA: New York Heart Association; LVEF: left ventricular ejection fraction; LVESV: left ventricular end-systolic volume; LVEDV: left ventricular end-diastolic volume; LA: left atrium; 6MWT: six-minute walking test; MLHQ: Minnesota Living with Heart Failure Questionnaire; SDNN: standard deviation of all normal R-R intervals recorded in a time interval; rMSSD: square root of the mean squared differences between adjacent normal R-R intervals over a time interval (milliseconds). Statistical tests used: unpaired Student’s t-test for symmetrical variables (displayed as mean ± SD) and Mann-Whitney test for asymmetrical variables [displayed as median (interquartile range)]. P values < 0.05 were considered significant.*

It was noted that SDNN in both groups before taVNS had similar levels, but analyzing Figure 3 , we can observe that, after the intervention, Group 1 reached better SDNN, and the same benefit was not observed in Group 2.

**Figure 3:** – Standard Deviation of R-R intervals (SDNN) before and after treatment. Statistical test performed: unpaired Student’s t. P values < 0.05 were considered significant.

When comparing the parameters before and after the intervention in intra-group analysis, we found that many improved significantly in Group 1, and there was no difference in Group 2 ( Figure 4 ). There was a benefit in Group 1 after taVNS regarding the quality of life, while there was not the same benefit in the *sham* group after 30 days of stimulation ( Figure 4 A). Likewise, superiority was observed for Group 1 in 6MWT when comparing before and after the intervention, something that did not occur in Group 2 ( Figure 4 B)

**Figure 4:** – Analysis of quality of life by Minnesota live with heart failure questionnaire – MLWHF – (A) and the 6-minute walk test (B) in groups 1 (taVNS) and 2 (sham) before treatment and after 30 days. In both cases, an improvement in these parameters was observed in the taVNS group (p<0.05) and no improvement in the sham group. Statistical tests performed: Wilcoxon-Mann-Whitney for quality of life and paired Student’s t-test for the 6-minute walk test. P values < 0.05 were considered significant.

We did not observe any complications or abandonment of treatment due to adverse events in our study. The summary of the design and findings of the study can be seen in Central Illustration .

**Central Illustration:** : Auricular Vagal Neuromodulation and its Application in Patients with Heart Failure and Reduced Ejection Fraction

Data analysis, and results. taVNS: transcutaneous auricular vagus nerve stimulation (intervention); EF: ejection fraction; sham: simulation (control); HR: heart rate; 6 min: 6 minutes.

## Discussion

This study showed that, in patients with HFrEF, when comparing stimulation with taVNS vs. *sham* , there was an improvement in the heart rate variability index in the intervention group, with no benefits in the other parameters. On the other hand, when intragroup variables were compared, there was an improvement in the 6MWT and MLHFQ after taVNS, while they did not change in the control group.

We have known since 1998, with the study by Nolan et al.^19 , 20^that the reduction in heart rate variability is an independent predictor of the increase in sudden death in HF and even in the general population.^21^We can suggest that taVNS, increasing HR variability, may be associated with a reduction of sudden death^21^by indirectly interfering in the reduction of the inflammatory cascade of HF, with less arrhythmic burden, through a better neurohumoral balance.

According to the HOPE4 HF^16^and BEAT HF,^24^using baroreflex activation therapy was safe and conferred benefits on HF. The present study demonstrated the same safety, ease of execution, and fewer side effects, in addition to showing benefits in HR variability and suggesting improvements in the 6MWT and quality of life. With the improvement in functional capacity, it was noticeable in all patients the desire to persist in treatment even during a pandemic and risks.

Frangos et al. in 2015^25^showed the benefit and ease of performing taVNS in humans in a non-invasive way, and it was possible to confirm in this study the same ease of execution.

Zannad and his group, in the NECTAR HF study,^17^failed to demonstrate the improvement of echocardiographic measurements after invasive VNS but demonstrated an improvement in quality of life. This finding could be replicated in our study without invasive intervention.

Improving the quality of life led to better adherence to treatment, lifestyle, perceptible patient satisfaction, greater engagement when noticing tangible results, and a new focus on their position regarding HF and their expectations.

Gold et al., in the INOVATE HF trial,^18^involving 85 centers, did not demonstrate a reduction in mortality when using invasive VNS, but a benefit in the 6-minute walk test, which is in line with our findings, with the advantage that we used non-invasive vagus nerve stimulation through the ear.

NYHA functional class and quality of life improved after VNS in several studies.^18 , 26^These positive effects demonstrated that most patients became less symptomatic and could better carry out day-to-day activities after treatment with VNS. The six-minute walk test was performed in five relatively recent studies, significantly increasing the distance walked in patients treated with VNS.^27 , 28^These findings align with the improvement in the 6MWT, and the quality of life observed in this study in the taVNS group, indicating that these patients became physically fitter after vagal stimulation. On the other hand, the present study could not demonstrate improvement in the NYHA functional class, probably because most patients were already in classes I or II from the beginning.

In the ANTHEM HF study,^29^Premchand et al. demonstrated that VNS on the left or right side of the neck had no difference in outcomes and was safe. In this study, it was decided to keep the stimulus in the right external ear by a simple convention.

In 2015 and again in 2020, in the TREAT AF study, Stavrakis et al.^30 , 31^demonstrated that taVNS suppressed and reduced the burden of atrial fibrillation in patients without HF, in addition to reducing the levels of pro-inflammatory cytokines. Recently, the same group demonstrated, in a pilot study, that taVNS reduced the levels of alpha tumor necrosis factor and improved the quality of life in patients with HF with preserved ejection fraction.^32^Our study hypothesizes that taVNS may also benefit patients with HF with reduced ejection fraction, as an improvement in heart rate variability was observed in the taVNS group.

Kaniusas and coworkers^33 , 34^systematically demonstrated the beneficial and anti-inflammatory effects of taVNS, not only through the classic mechanisms exposed in their studies but also through others still poorly understood. This study generates a hypothesis by demonstrating that we obtained promising results in heart failure by modulating excess sympathetic activity and stimulating parasympathetic activity.

In a recent publication, Sant’Anna et al.^8^performed a meta-analysis on randomized clinical studies comparing invasive VNS plus drug treatment vs. drug treatment in HF and observed that in patients with HFrEF, the use of VNS was associated with improvement in NYHA functional class, quality of life, 6MWT and reduction in NT-proBNP levels. This study showed improvement in heart rate variability, quality of life, and 6MWT, with fewer adverse effects than invasive studies that used implantable devices.

### Limitations

This study had some limitations:

1. Group 1 had, in the pre-intervention phase, higher age, worse quality of life, and higher rMSSD than Group 2, which may impair the analysis after the intervention. We attributed this finding to the small sample size, but the results showed that such discrepancies did not influence the final findings.
2. The COVID-19 pandemic was an obstacle to carrying out this study. The concern of patients for having heart disease and the risk of contagion is highlighted. Such obstacles were overcome by changing the environment and informing that protective measures would be provided, although this did not affect the data analysis.
3. Another limitation brought about by the pandemic was the economic crisis, making it difficult to mobilize to carry out treatment and serial examinations. We provide tickets, food allowance, and fundamental clarification on the importance of treatment.
4. We did not measure biomarkers in this study as, until the time of recruitment, we did not have a laboratory with such resources available in our region. However, the original idea was to generate a hypothesis for outpatient treatment, which was done.
5. An important limitation stems from the short term of the study. Most studies of vagal stimulation have shown a more noticeable result after a longer stimulation period, whereas the treatment period in this study was only 1 month. As the results were still promising, new studies are expected to clarify the minimum and ideal time soon to obtain a reasonable effect of vagal modulation in HF.
6. Another limiting factor was the NYHA functional class of the patients, most were class I (27.9%) or II (39.5%), and therefore the objective of evaluating the improvement in functional class in these patients lost its meaning. New studies involving the use of taVNS in the treatment of HF should exclude NYHA class I since the desired clinical benefit has already been achieved in these patients.

## Conclusion

taVNS is a safe, easy-to-perform intervention and can benefit HF by improving heart rate variability parameters (SDNN), which indicates better autonomic balance. In intragroup comparisons before and after treatment, improved quality of life and the 6-minute walk test in the taVNS group were also shown.

Based on these results, one can suggest expanding the indication of auricular vagal neuromodulation in patients with HF, although new studies with a larger number of patients are needed to answer the questions raised by the present study.
